# Recycling Blast Furnace Ferronickel Slag as a Replacement for Paste in Mortar: Formation of Carboaluminate, Reduction of White Portland Cement, and Increase in Strength

**DOI:** 10.3390/ma14102687

**Published:** 2021-05-20

**Authors:** Qingfeng Guan, Jingliang Xia, Jing Wang, Faguang Leng, Yongxiang Zhou, Changwei Cao

**Affiliations:** 1 Institute of Building Materials, China Academy of Building Research, Beijing 100013, China; guan_qf@126.com (Q.G.); xiasnxia@126.com (J.X.); wangking3007@126.com (J.W.); lengfaguang@cabrtech.com (F.L.); 2 National Engineering Research Center of Building Technology, Beijing 100013, China; 3 State Key Laboratory of Building Safety and Built Environment, Beijing 100013, China; 4 China Road and Bridge Co., Beijing 100011, China; legercao@126.com

**Keywords:** ferronickel slag waste, strength, carboaluminate, ettringite stabilization, carbon footprint

## Abstract

Blast furnace ferronickel slag (BFFS) is generated in the production of ferronickel alloys and is used as cement replacement in concrete or mortar. The effectivity in reducing cement consumption and improving performance are limited. By referring to the paste replacement method, this work used BFFS to replace an equal volume of the white Portland cement paste to obtain greater performance enhancement. BFFS was used with five levels of replacement (0%, 5%, 10%, 15%, 20%) and four water-to-cement ratios (0.40, 0.45, 0.50, 0.55) were designed. Fluidity, mechanical strength, hydration products, and pore structure of every mixture were measured. The results showed that the workability of the mortars decreased due to the reduced volume of water, but the 28-day compressive strength of the mortars increased, and the cement content of the mortars was also reduced by 33 wt %. The X-ray diffraction (XRD) patterns revealed that there existed a carboaluminate phase, and the presence of the ettringite was stabilized, indicating that the accumulating amount of the hydration products of the mortar increased. Furthermore, the BFFS could consume the portlandite and free water to form a higher amount of chemically bound water due to its pozzolanic activity. A high degree of hydration and a large volume of the hydration products refined the porosity of the hardened mortars, which explained the enhancement of the strength of the mortars. Compared to the cement replacement method, the paste replacement method was more effective in preparing eco-friendly mortar or concrete by recycling BFFS for reducing the cement content of the mortar while improving its strength.

## 1. Introduction

As a type of waste residue, ferronickel slag has gradually attracted increasing attention in recent years [1,2]. In China, the annual production of the ferronickel slag reaches 40 million tons and accounts for 20% of the total production of the metallurgical slag; it is the fourth most produced byproduct material after blast furnace slag, steel slag, and red mud [3]. Moreover, in South Korea, the production of ferronickel slag is 2 million tons annually [4,5]. This huge amount of ferronickel slag is usually dumped on the landfill and may cause heavy metal contamination in the disposal site, thereby creating serious environmental issues and health problems [6]. In terms of the smelting process, ferronickel slag can be classified as the blast furnace ferronickel slag (BFFS) and the electric furnace ferronickel slag (EFFS) [1]. In the production of ferronickel alloys, iron and nickel are extracted from the nickel ore at a temperature of approximately 1600 °C, and the waste residue is then quenched by water to form BFFS [3]. Consequently, BFFS consists of an amorphous phase and mainly contains SiO_2_, MgO, Fe_2_O_3_, CaO, and Al_2_O_3_ [2,7]. Actually, EFFS has been researched as the fine aggregate [8,9,10], as the raw material to produce cement clinker [11], as a supplementary cementitious material (SCM) [1,12], and as a geopolymer [13,14], but the investigations into BFFS have largely focused on its application as a supplementary cementitious material and need to be further promoted. 

Some major bridges such as landmark cable-stayed bridges and suspension bridges are expected to have white and clean bridge towers constructed chiefly of white Portland cement (WPC). On the other hand, there are strict requirements for the selection of raw materials [15], the calcination temperature, and fineness of particles during the production of WPC [16]. Moreover, producing the WPC massively consumes a great deal of energy and emits a huge amount of carbon dioxide; meanwhile, the cost of construction increases by the high price of the WPC [17]. According to the traditional strategy which was named the “cement replacement method” by Li et al. [18], the WPC content of mortar or concrete can be reduced by the proper addition of the SCMs without noticeable adverse effects on the mechanical performance and durability of mortar or concrete. In this strategy, the SCM is generally added to replace part of cementitious materials [19,20,21,22]. Nonetheless, at a high rate of cement replacement, the overall performance of mortar or concrete is substantially reduced, so the effectiveness of supplementary cementitious materials in reducing the WPC content of the mortar or concrete and carbon footprint will be limited in the cement replacement method. In addition, nonwhite mineral admixtures will affect the aesthetic appearance of white concrete structures. To decrease the consumption of WPC while simultaneously maintaining the performance of cement-based materials unchanged, an innovative replacement method is desired. 

In recent years, Li et al. [23,24,25] developed an alternative technology named the “paste replacement method” which recycles waste residues or inert fillers to replace an equal volume of cement and water in a mortar paste at an unchanged mix proportion. Consequently, the water-to-cement ratio can remain constant although the substitution rate of the cement is gradually increased. The relevant findings of Li et al. [26,27,28] demonstrated that the strength, dimensional stability, and durability of the mortar produced by the paste replacement method somewhat improved. Nevertheless, they did not explain the mechanism whereby the paste replacement method affects the properties of mortar or concrete from the perspective of the hydration process. 

To increase the addition of BFFS and reduce the consumption of WPC, the present work compared the paste replacement method with the cement replacement method through producing a series of mortar mixes with various amounts of BFFS and different water-to-cement ratios and analyzing the strength, workability, and microstructure of the resultant mortars. Moreover, we evaluated the effectiveness of the two replacement methods in the reduction of cement consumption, the improvement in the strength of the mortar, the densification of the mortar microstructure, and waste utilization.

## 2. Materials and Methods

### 2.1. Raw Materials

CEM I 52.5R white Portland cement, produced in Tunisia, with a loss on ignition of 2.1%, a specific gravity of 3.077, and a specific surface area of 334 m^2^/kg, was used to produce the WPC mortar. According to Table 1, listing the chemical composition of the WPC determined by the X-ray fluorescence spectrometry (XRF), it is chiefly composed of CaO (72.27%), SiO_2_ (18.77%), and Al_2_O_3_ (3.98%). The results of the Rietveld refinement of X-ray diffraction (XRD) show that tricalcium silicate (C_3_S), dicalcium silicate (C_2_S), tricalcium aluminate (C_3_A), and tetra-calcium aluminoferrite (C_4_AF) respectively constitute 53.29%, 29.92%, 3.76%, and 0.38% of cement, while the cement contains 8.8% calcite. The moisture content, water absorption, specific gravity, and maximum particle size of the natural sand selected as the fine aggregate were measured to be 0.13%, 0.91%, 2.665, and 1.18 mm, respectively. 

**Figure 1:**
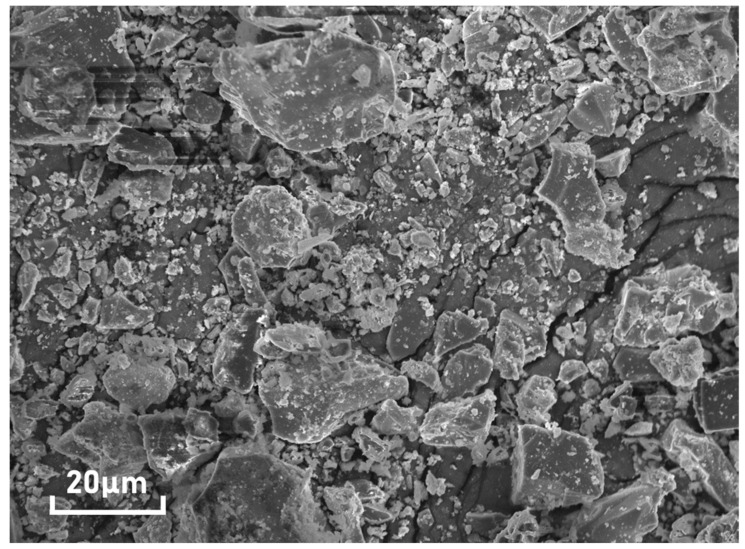
The SEM image of the blast furnace ferronickel slag.

Obtained from Fujian province in China, the original BFFS with an angular appearance (Figure 1) investigated herein was a dry powder ground from the iron-extracted waste which was first quenched with water and then dried. Its chemical components, as given in Table 1, primarily consist of CaO (31.73%), SiO_2_ (26.85%), and Al_2_O_3_ (20.92%). Moreover, the specific gravity, maximum particle size, and specific surface area of the BFFS were measured to be 2.855, 0.15 mm, and 384 m^2^/kg, respectively.

A polycarboxylate-based superplasticizer (SP), purchased from Subote New Materials Ltd., Nanjing, China, in the form of a white powder was used in the mixing process of the mortars to achieve the constant workability. Generally, a mortar mix with a lower water-to-powder ratio (the total volume of the WPC and the BFFS) needs a higher dosage of the superplasticizer to disperse the powder particles.

### 2.2. Mix Proportions

In the paste replacement method, 20 mortar mixes with various volumes of the BFFS as a replacement for the mortar paste and a series of water-to-cement ratios (W/C) were designed and investigated. Firstly, the water-to-cement ratios were designed as 0.40, 0.45, 0.50, and 0.55 by mass. It should be noted that the W/C remained constant although the BFFS was incorporated into the mortar mixes to reduce the WPC content of the mortar. 

The total volume of the paste of all the mortar mixes, that is, the total volume of water, the WPC, and the BFFS as a percentage of the mortar volume, was maintained at 60%, and the volume of the fine aggregate was set at 40%, as listed in Table 2. To maintain the total volume of the mortar paste unchanged, the volume of the cement paste, i.e., the volume of the WPC and water calculated as a percentage of the total volume of the mortar, was reduced correspondingly by the incorporated volume of the BFFS. More precisely, the volume of the BFFS was set at 0%, 5%, 10%, 15%, and 20%, and the corresponding volume of the cement paste was 60%, 55%, 50%, 45%, and 40%. Then, the dosage of the superplasticizer, that is, the mass of the superplasticizer powder expressed as a percentage of the total mass of the WPC and the BFFS, was not preset since the superplasticizer needed to be added little by little to the mortars to achieve an expected flow spread in the range of 180 to 350 mm.

In addition, the experiments were designed to compare the difference between utilizing the BFFS as a replacement for the cement and using it as a replacement for the paste as described above by preparing another eight mortar samples. Similarly, in the cement replacement method, the total volume of the paste of each mix proportion was set at 60%, and the percentage of the fine aggregate remained unchanged at 40%. The volume of the BFFS varied from 0% to 20% with increments of 5%, while the corresponding volume of the cement paste decreased from 60% to 40% with decrements of 5%. It should be emphasized that the incorporation of the BFFS as a replacement for the cement did not change the volume of water but decreased the volume of the WPC, indicating that the actual W/C changed. Moreover, only the water-to-cement ratios of 0.40 and 0.55 were taken into account in this investigation. 

As presented in Table 2, a fixed sample ID of X-Y-Z was employed to identify each mortar mix, in which X shows the type of the mortar: PRB, which represents the mortar with different volumes of the BFFS incorporated as a substitute for the paste, and CRB, which indicates the mortar with different volumes of the BFFS incorporated as a substitute for the cement; Y stands for the W/C; and Z denotes the percentage of the incorporated BFFS.

### 2.3. Testing Methods

#### 2.3.1. Particle Size Distribution of Raw Materials 

The particle size distributions (PSD) of the powder materials (the WPC and the BFFS) and the fine aggregate were measured by a laser particle size analyzer and a mechanical sieving machine, respectively. 

#### 2.3.2. Workability of Mortar

Similar to the slump flow test of concrete [29], this study used a small-sized slump cone test [30] to measure the slump flow of each mortar specimen listed in Table 2. Having been filled up with the fresh mortar, the mini slump cone was lifted vertically, and then the mortar slumped and flew in a patty shape. Finally, the average of the two diameters in the orthometric direction of the patty was considered to be the slump flow of the mortar sample.

#### 2.3.3. Flexural Strength and Compressive Strength of Mortar

According to Chinese standard JGJ/T 70-2009 [31], three prismatic specimens with the dimensions 40 mm × 40 mm × 160 mm of each batch of the fresh mortar mixture were cast and cured in a standard curing room at a temperature of 23 ± 2 °C and relative humidity of greater than 95% after the mini slump cone test. At a curing period of 28 days, the flexural strength of the mortars was first tested, and the compressive strength test was conducted on the half of the prismatic specimens fractured in the flexural strength test. For both the flexural strength and compressive strength of the mortars, the average value of the three prismatic specimens measured and under similar conditions was regarded as the final value. 

#### 2.3.4. Hydration Products and Pore Structure of Mortar

To characterize the microstructure of the paste containing different volumes of the BFFS, the samples listed in Table 2 were used to prepare the paste samples without the fine aggregate and then cured at a temperature of 23 ± 2 °C and relative humidity of greater than 95%. After 28 days, these paste samples were cut into slices and soaked in anhydrous ethanol for 5 days to stop hydration; ethanol was also renewed after 24 h. Finally, the slices were removed from the ethanol and dried in a vacuum drying chamber at a temperature of 40 °C for 7 days to remove the ethanol [32].

The mineral composition of the raw materials and the hardened pastes was measured at the tested curing periods by the X-ray diffraction measurement (D/MAX-IIIB) using a CuKα source operated at 40 mA and 40 kV, at a 2θ angle ranging from 5° to 70°, and at a scan rate of 5°/min. 

The thermogravimetric analysis (TGA) (STA8000, Perkin Elmer, Waltham, MA USA) of the paste specimens of about 15 mg was conducted in a temperature range of 30 to 1000 °C at a heating rate of 10 °C/min under a 30 mL/min constant flow of N_2_ to measure the amount of portlandite (CH) and chemically bound water. 

Approximately 1 g pieces from the inner part of the different hardened pastes were used to characterize the pore structure of the mortars by means of mercury intrusion porosimetry (MIP) (PoreMaster, Quantachrome, USA) in a high-pressure mode from 140 kPa to 230 MPa and at a contact angle of 140° between the paste and the mercury.

## 3. Results

### 3.1. PSD and XRD Patterns of Raw Materials

**Figure 2:**
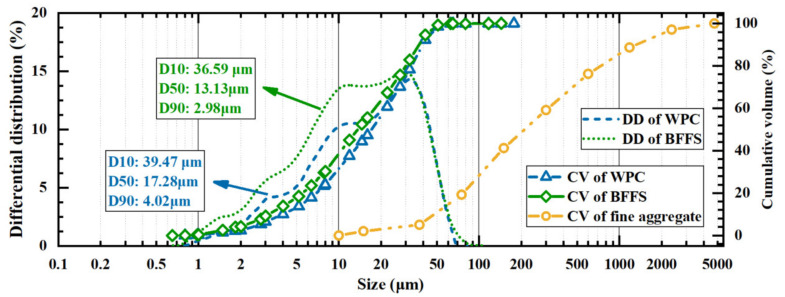
The particle size distributions of the white Portland cement, the blast furnace ferronickel slag, and the fine aggregate; DD: differential distribution; CV: cumulative volume.

The particle size distributions of the WPC, the BFFS, and the fine aggregate measured by the laser particle size analyzer are shown in Figure 2. The WPC and the BFFS both had a continuous graded particle size distribution. Moreover, the size of the WPC varied from 0.80 to 50.89 μm, and its average particle size was 17.28 μm, which was larger than the average particle size of the BFFS (13.13 μm) utilized in this experiment.

**Figure 3:**
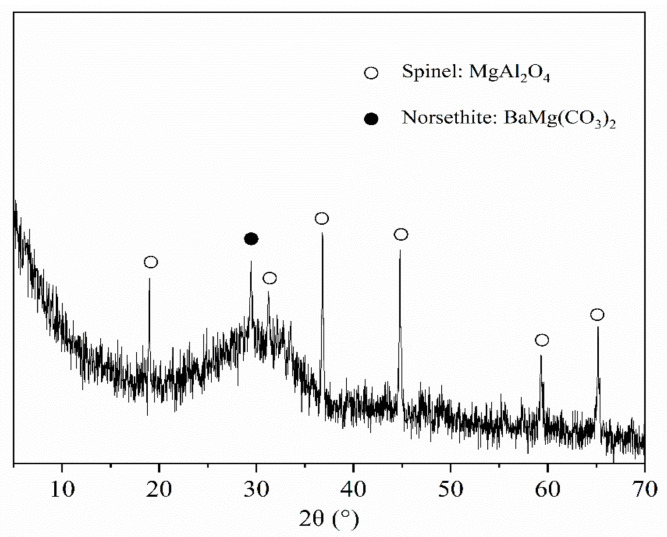
The X-ray diffraction pattern of the BFFS powder.

From the XRD pattern presented in Figure 3, we could infer that the mineral composition of the BFFS chiefly consisted of spinel (MgAl_2_O_4_) and norsethite (BaMg(CO_3_)_2_), indicating that although the MgO content of the BFFS reached as high as 9.2% (see Table 1), it did not cause volume expansion in the mortar mixture because the MgO in the BFFS acted as an inert mineral in the forms of spinel (MgAl_2_O_4_) and norsethite (BaMg(CO_3_)_2_). Moreover, since most of the BFFS is produced by water quenching, its mineral components are dominated by an amorphous phase and exhibit certain pozzolanic activity. Previous investigations [1,33] also demonstrated that the ferronickel slag recycled for producing concrete did not cause heavy metal pollution since its corrosivity, radioactivity, and amount of toxic leachate were lower than the maximum allowable value.

### 3.2. Workability

**Figure 4:**
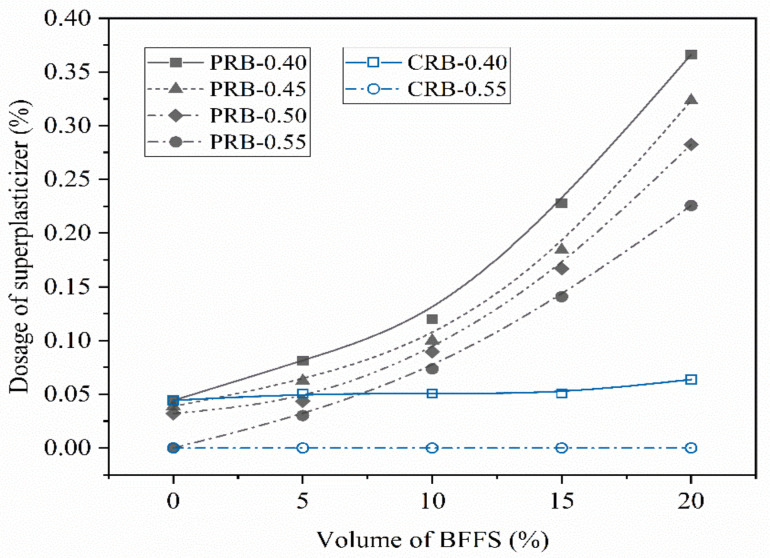
The variation in the dosage of the superplasticizer of the mortar with the volume of the BFFS at various volumes of the replaced paste (PRB) and the replaced cement (CRB).

**Figure 5:**
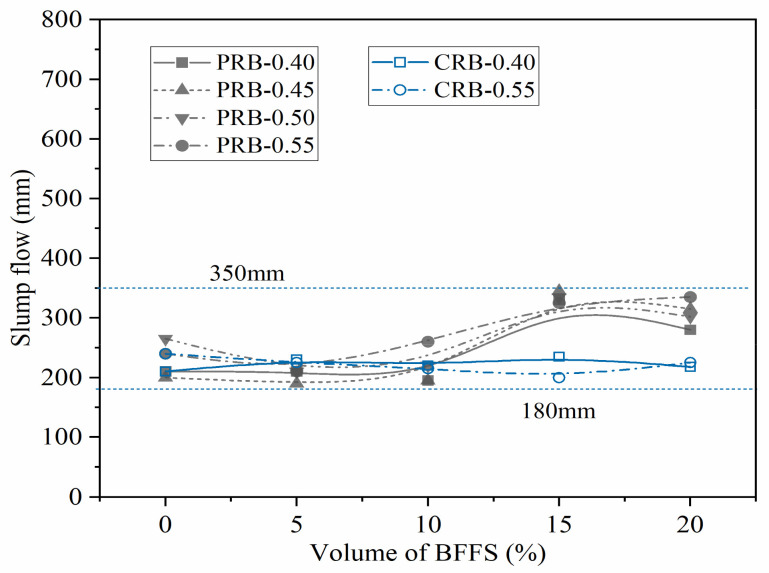
The variation in the slump flow of the mortar.

The data in the sixth column of Table 2 list the dosage of the superplasticizer required for the mortar mixture to obtain the expected flow spread in the range of 180 to 350 mm. Figure 4 shows the dosage of the superplasticizer versus the volume of the BFFS for both the PRB and CRB mortars; the slump flow of each sample is also listed in the second column of Table 3 and is illustrated versus the volume of the BFFS for both the PRB and CRB specimens in Figure 5. It is clear that the slump flow of the mortar specimens varied from 195 to 345 mm and showed slight fluctuations.

As shown in Figure 4, when the volume of the BFFS remained constant, a higher W/C generally decreased the dosage of the superplasticizer. When the W/C remained constant, the replacement method of the BFFS significantly affected the dosage of the superplasticizer. For example, at a W/C of 0.40, in the paste replacement method, the dosage of the superplasticizer increased from 0.04% to 0.37% as the volume of the BFFS was raised from 0% to 20%, while, in the cement replacement method, the dosage of the superplasticizer marginally increased from 0.04% to 0.06% as the volume of the BFFS was raised from 0% to 20%. This indicates that the incorporation of the BFFS as a replacement for the paste required a higher dosage of the superplasticizer than its incorporation as a replacement for the cement.

### 3.3. Mechanical Strength of Mortar

The third to sixth columns of Table 3 present the data on the 3-day and 28-day flexural and compressive strength of the mortar specimens. At a given volume of the BFFS, increasing the W/C reduced both the flexural strength and the compressive strength of the mortar, which was reasonable since the W/C played a crucial role in the development of mortar strength; a higher W/C generally resulted in a mortar with a lower strength. 

Another noteworthy phenomenon was that at a given W/C, the variation in the compressive strength of the PRB mortar specimens was different from that of the CRB mortar specimens. However, at the same W/C, when the BFFS was added as a substitute for the cement, raising the volume of the BFFS from 0% to 20% reduced the 3-day flexural strength of the mortar. Similarly, when the BFFS was added as a substitute for the paste, increasing the volume of the BFFS from 0% to 20% improved the 28-day flexural strength and compressive strength; nevertheless, when the BFFS was added as a substitute for the cement, increasing the volume of the BFFS from 0% to 20% reduced the 28-day flexural strength of the mortar and increased the compressive strength of the mortar. The other mortar samples with different water-to-cement ratios also showed a similar variation in the flexural strength and compressive strength after a curing period of 3 or 28 days.

**Figure 6:**
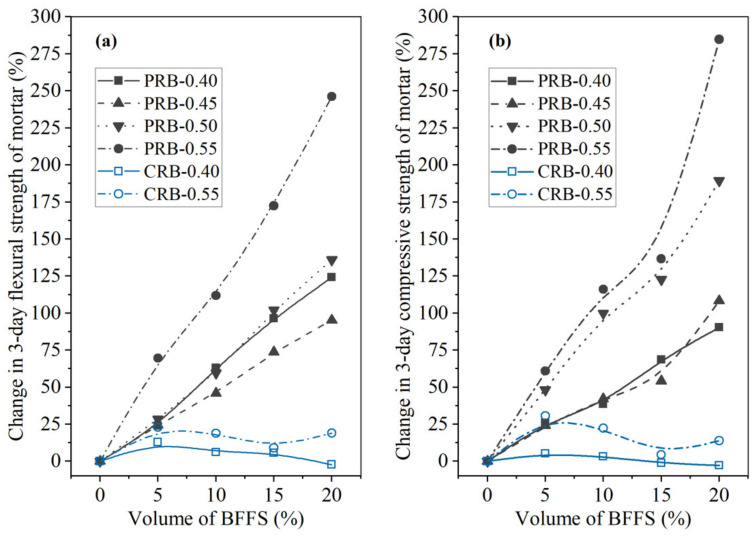
The percentage of the change in the 3-day strength of the mortar versus the volume of the BFFS: (**a**) flexural strength; (**b**) compressive strength.

**Figure 7:**
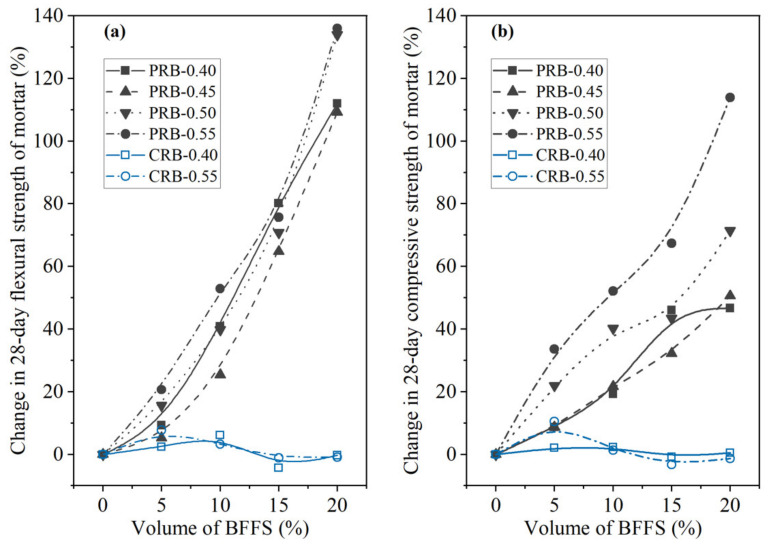
The percentage of the change in the 28-day strength of the mortar versus the volume of the BFFS: (**a**) flexural strength; (**b**) compressive strength.

To further analyze the effectiveness of the two strategies for applying the BFFS to the mortar, that is, the paste replacement method and the cement replacement method, in the enhancement of the strength of the mortar, the percentage of the change in the flexural strength and the compressive strength of the mortars cured for 3 and 28 days as a function of the volume of the incorporated BFFS are presented in Figure 6 and Figure 7. Regardless of the curing period, the percentage of the change in the flexural strength and compressive strength of the mortar increased at any given water-to-cement ratios when the BFFS was added to substitute an equal volume of the paste. Meanwhile, the improvement to the percentage of the change in the strength of the mortar was more remarkable as the W/C increased. For example, the change in the 28-day flexural strength and compressive strength of sample PRB-0.40-20 was 112.0% and 46.7%, respectively, while that of sample PRB-0.55-20 was 135.9% and 113.9%, respectively. The data on the 3-day flexural strength and compressive strength of the mortar also followed a similar changing pattern. By contrast, when the BFFS was added to replace the cement, the percentage of the change in the strength of the mortar slightly increased at 5 vol% of the BFFS but then declined as the volume of the BFFS increased further. With respect to the 28-day strength of the mortar, the maximum volume of the BFFS had to be smaller than 10% when it was utilized as a replacement for the cement, which was at least 10% lower than when it was used as a replacement for the paste. Therefore, the paste replacement method could provide a positive effect.

### 3.4. Hydration Process

#### 3.4.1. Phase Composition 

**Figure 8:**
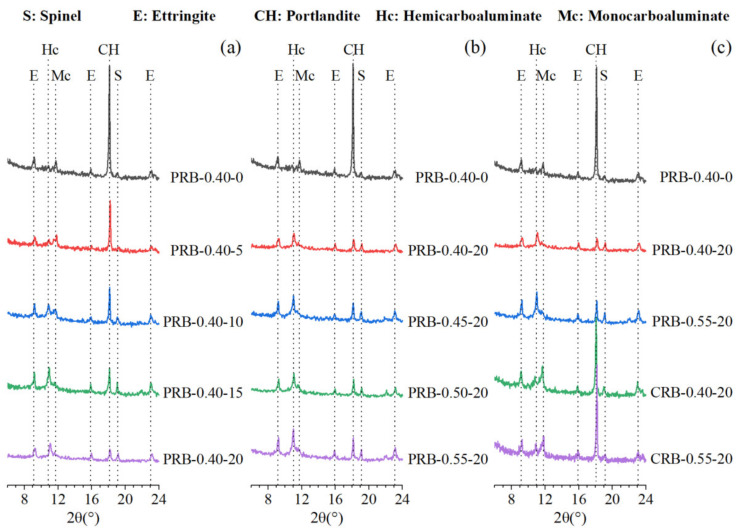
The XRD patterns of the paste containing the BFFS after 28 days of hydration: (**a**) the pastes produced by the paste replacement method at a water-to-cement ratio of 0.40 and different volumes of the BFFS; (**b**) the pastes produced by the paste replacement method with 20 vol% of the BFFS at different water-to-cement ratios; (**c**) the pastes produced by different replacement methods with 20 vol% of the BFFS.

The 28-day hydration products of the mortar samples at different water-to-cement ratios and various volumes of the BFFS were characterized by the XRD as shown in Figure 8. All the samples in Figure 8 formed portlandite and ettringite after the hydration process, and there was inert spinel remaining in the cementitious systems. When the volume of the BFFS increased, the hydration process gradually produced a considerable amount of hemicarboaluminate (Hc) and monocarboaluminate (Mc). In general, when the cementitious system contains a high amount of calcite and amorphous alumina simultaneously, the hydration process might consume the portlandite and water to form a carboaluminate phase [34]. It can be concluded from Table 1 and Figure 3 that the BFFS contained a large amount of amorphous alumina, and the WPC consisted of 8.8% calcite, indicating that the mortar samples containing the BFFS satisfied the conditions required for the hydration process, that is, they had the necessary reactants. By contrast, previous investigations [3,12] have reported that electric furnace ferronickel slag largely consists of crystalline minerals, including forsterite and enstatite, and contains basically no amorphous alumina, so it cannot be tested for the diffraction peaks of the carboaluminate phase when it reacts with Portland cement.

Figure 8a shows that as the volume of the BFFS increased, the dominant carboaluminate phase gradually changed from the Mc to the Hc. As a result of the addition of the BFFS, the WPC content of the mortar, as well as the amount of the calcite of the mortar, decreased, but the volume of the Al phase increased. At a higher bulk ratio of CO_2_ to Al_2_O_3_, the hydration process appeared to be more inclined to form the hemicarboaluminate after 28 days of hydration. This phenomenon was also observed in other investigations [35,36]. A similar phase composition was also seen in the mortar with a higher W/C, as shown in Figure 8b. Li et al. [2] used Portland cement and ferronickel slag with only 6.56% Al_2_O_3_ to prepare mortar and found the existence of the monocarboaluminate after 3 and 240 days of hydration, but the diffraction peaks of it were indistinguishable due to the limited amorphous Al content of the ferronickel slag. Kim et al. [37] utilized superfine ferronickel slag with abundant MgO and Al_2_O_3_ as a replacement for the cement and discovered a high amount of hydrotalcite (Mg_6_Al_2_(CO_3_)(OH)_16_·4H_2_O) by the XRD measurement.

In addition to the carboaluminate phase, the hydration process of the paste containing the BFFS could consume the portlandite to form secondary hydration products, such as calcium (aluminum) silicate hydrate (C-(A)-S-H) gels, C_4_AH_13_, C_3_AH_6_ (hydrogarnet), and strätlingite because the BFFS was an alumina-rich pozzolanic material and could react with the portlandite. Strätlingite as a type of a monosulfate (Ms) phase is a typical product with a hexagonal structure and crystal composition of C_2_ASH_8_ in the cementitious pastes with aluminate-rich pozzolanic materials; it has also been reported that it coexists with the sulfate phase, ettringite, and carboaluminate. However, the peak of strätlingite, usually appearing at a 2θ angle of 7.2°, is absent in Figure 8, which might potentially be attributed to the existence of the portlandite. From a thermodynamic perspective [38], strätlingite cannot be compatible with the portlandite unless the amount of Portland cement is depleted at later stages [35]. 

Meanwhile, the diffraction peak of the ettringite in Figure 8 was clearly detected since the available CO_3_^2−^ dissolved from the calcite could restrain the transformation of the ettringite into the Ms; thus, it indirectly stabilized the existence of the ettringite although the gypsum as the source of sulfate was depleted [32,39]. This chemical process respectively increased the bulk of the total hydration products and thus was beneficial to refining the microstructure and increasing the strength of the mortar [38].

Compared to the PRB samples, the samples prepared by replacing the cement with the BFFS experienced the formation of the carboaluminate phase, but the dominant phase was the monocarboaluminate rather than the hemicarboaluminate. Similar to Figure 8c, the WPC content of the CRB samples was higher than that of the PRB specimens at the same volume of the BFFS, so the amount of the calcite was higher; therefore, the transformation of the hemicarboaluminate into the monocarboaluminate was promoted.

#### 3.4.2. Thermogravimetric Analysis

**Figure 9:**
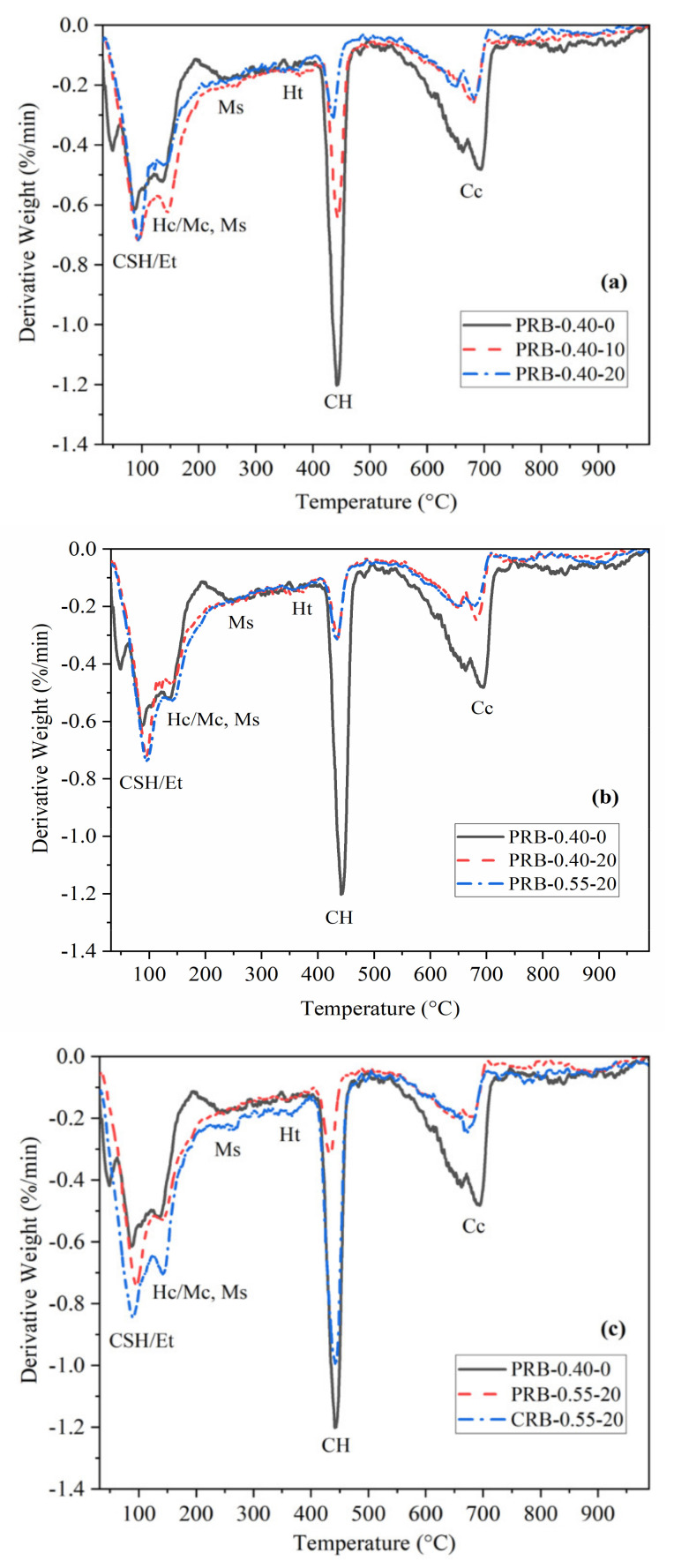
The differential thermal analysis curves of the pastes after 28 days of hydration: (**a**) the pastes produced by the paste replacement method with different volumes of the BFFS; (**b**) the pastes produced by adding 20 vol% of the BFFS to replace an equal volume of the paste at different water-to-cement ratios; (**c**) the pastes produced by different replacement methods with 20 vol% of the BFFS; CSH: calcium silicate hydrates; Et: ettringite; Hc: hemicarboaluminate; Mc: monocarboaluminate; Ms: monosulfate; Ht: hydrotalcite; CH: Portlandite; CO_3_^2−^: carbonate.

In this section, the results of the thermal analysis of the mortar were discussed mainly through examining the thermogravimetric analysis (TGA) curves. As shown in Figure 9, these curves largely consisted of three major peaks. The first peak up to a temperature of 400 °C indicated the dehydration reaction of the calcium silicate hydrate (C-S-H) gels, monosulfate phase, carboaluminate phase, and ettringite; the second major peak in a temperature range of 400 to 525 °C, in a high-pressure mode, was relevant to the dehydroxylation of the portlandite; the last peak, approximately in a temperature range of 525 to 750 °C, in a high-pressure mode, resulted from the decarbonation of the carbonates to emit CO_2_. The range of the temperature of these three major peaks may be different from what is reported in other works [40,41] due to the difference of the instrument parameters, the gaseous atmosphere of the testing, the heating rate, and the preparation of the dried samples, but the amount and order of the major peaks are the same. In addition, all the pastes showed a slight mass loss of hydrotalcite (Ht) at a temperature of approximately 380 °C, which was possibly caused by the formation of Mg-containing hydrates resulting from the presence of magnesium in the clinker, particularly in the BFFS. However, there was no XRD peak related to the hydrotalcite observed in Figure 8 owing to its limited amount and poor crystallinity. 

Further, the thermodynamic stability of the Ms was slightly higher than that of the Hc–Mc since its decomposition peak was higher than that of the carboaluminate phase. This difference mainly originated from the fact that the temperature of the dehydration of the octahedral layer of the Ms was slightly higher than that of the Hc–Mc [42]. When the samples contained the BFFS, no distinct peak of the Ms was detected in a temperature range of 200–300 °C in Figure 9, and the peaks in a temperature range of 140–165 °C were lower than those of the pure cement, which demonstrated that the ettringite stabilization was confirmed by the XRD results due to the existence of CO_3_^2−^. Moreover, the consumption of the portlandite owing to the addition of the BFFS was observed as a gradually decreasing peak in a temperature range of 400–525 °C; correspondingly, the peaks of the C-S-H and the ettringite were enhanced, but the peak of the decarbonation weakened due to the higher degree of hydration.

**Figure 10:**
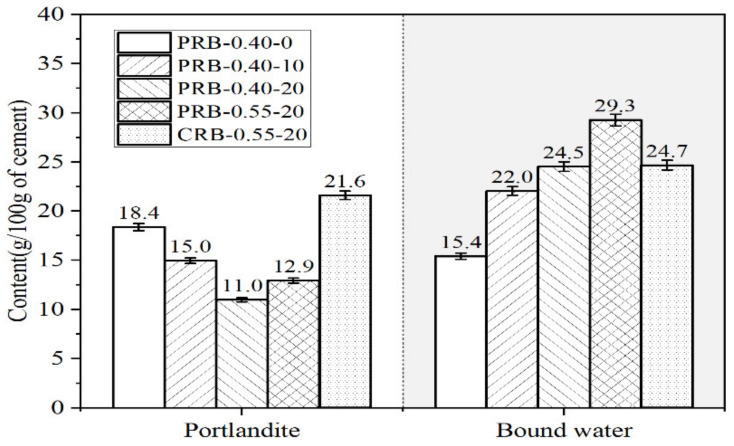
The amounts of the portlandite and bound water of the samples produced by the paste replacement method or the cement replacement method with different volumes of the BFFS, at various water-to-cement ratios, and at a curing period of 28 days.

The amounts of the portlandite and bound water of the pastes with different volumes of the BFFS and various water-to-cement ratios are shown in Figure 10. The tangential step was used to determine the weight loss of the portlandite in a temperature range of 400–525 °C, and the horizontal step was employed to determine the weight change between 30 and 525 °C. The drying weight at a temperature of 525 °C was assumed to be the solid content of the paste and was used to normalize the portlandite content and the amount of the bound water (H) per 100 g of the cement according to Equations (1) and (2) [39,42]:

W_H_ = (W_30_ − W_525_)/W_525_ × 100/(% cement) (1)

W_CH_ = (W_400_ − W_525_)/W_525_ × (74/18) × 100/(% cement) (2)

At a fixed W/C of 0.40, the portlandite content per 100 g of the cement decreased as the volume of the BFFS increased, and the percentage of its reduction rose at a higher replacement level of the BFFS. This change in the portlandite content could be explained by the pozzolanic reaction of the BFFS and the formation of the Hc and the Mc. It should be noted that, in the paste replacement method, the dilution effect does not exist since the water-to-cement ratio by mass remains unchanged when the BFFS is added. Thus, in the case of the addition of the BFFS, the normalized amount of the bound water increased correspondingly. In addition to the pozzolanic activity of the BFFS, the nucleation effect of the finer particles of the BFFS was possibly another main factor which could enhance the hydration process of the clinker with enough free water during a hydration period of 28 days to form a higher amount of the hydration products; however, the dilution effect should not be taken into account.

**Figure 11:**
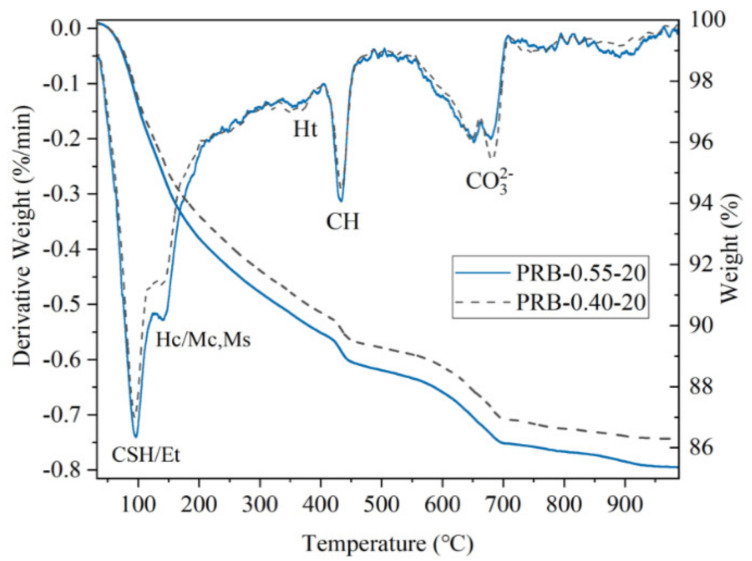
The TGA and DTA curves of pastes PRB-0.40-20 and PRB-0.55-20 at a curing period of 28 days.

In the case of adding 20 vol% of the BFFS, the amounts of the portlandite and bound water of the paste with a high W/C (such as 0.55) were higher than those of the paste with a low W/C (such as 0.40), indicating that the degree of the hydration of sample PRB-0.55-20 was higher, and more hydration products were formed due to the higher amount of the free water. According to the differential thermal analysis (DTA) curves in Figure 11, in addition to the higher peaks of the portlandite, the C-S-H, and the ettringite (CSH–Et) in sample PRB-0.55-20, the mass loss of the carboaluminate phase (Hc–Mc) was distinctly higher, while the peak associated to the decarbonation of the carbonate was weaker. The adequate free water in sample PRB-0.55-20 not only could enhance the hydration of the anhydrous clinker but also could accelerate the chemical reaction between the amorphous alumina of the BFFS, the portlandite, the hydration products of the clinker, and the calcite of the WPC. In the paste of sample PRB-0.40-20, the factual water-to-powder ratio by weight (where the powder consisted of the WPC and the BFFS) in Table 2 was reduced to approximately 0.19 due to the 20 vol% addition of the BFFS. A severe water shortage leads to an intense competition for water among different hydration processes. In such a case, first, the hydration of the clinker with the highest reactivity is restricted. Then, the secondary hydration such as a pozzolanic reaction and additional reactions forming aluminates and the carboaluminate phase are also hindered, which causes the amounts of the portlandite and bound water of sample PRB-0.40-20 to be lower than those of sample PRB-0.55-20 (see Figure 10) and leads to a lower mass loss as shown in the TGA curves in Figure 11.

Adding the BFFS to only replace an equal amount of the cement produced the dilution effect, a higher amount of the anhydrous clinker participated in the hydration reaction. Therefore, the portlandite content of sample CRB-0.55-20 in Figure 10 significantly increased at the same curing period. Nonetheless, compared to sample PRB-0.55-20 prepared by the paste replacement method, sample CRB-0.55-20 contained a lower mass fraction of the BFFS, which potentially resulted in a relatively low degree of secondary hydration and a lower amount of bound water at a curing period of 28 days.

#### 3.4.3. Porosity Refinement

**Figure 12:**
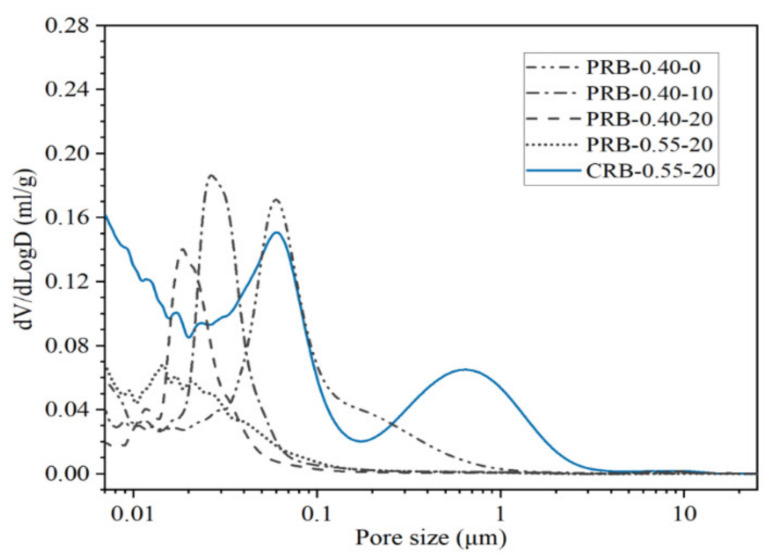
The pore size distribution of the pastes after 28 days of hydration.

**Figure 13:**
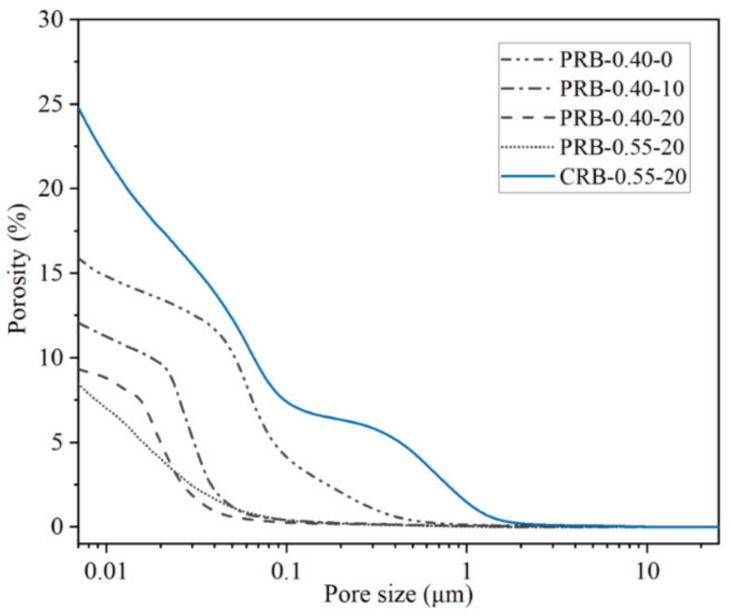
The porosity of the pastes after 28 days of hydration.

The results of the mercury intrusion porosimetry in terms of the pore size distribution and porosity of the pastes at a curing period of 28 days are shown in Figure 12 and Figure 13. Adding the BFFS as a replacement for the paste could reduce the porosity and the volume fraction of large pores while this refinement was more effective at a high W/C. When the BFFS was incorporated as a replacement for the cement, the pastes still had more large pores. 

**Figure 14:**
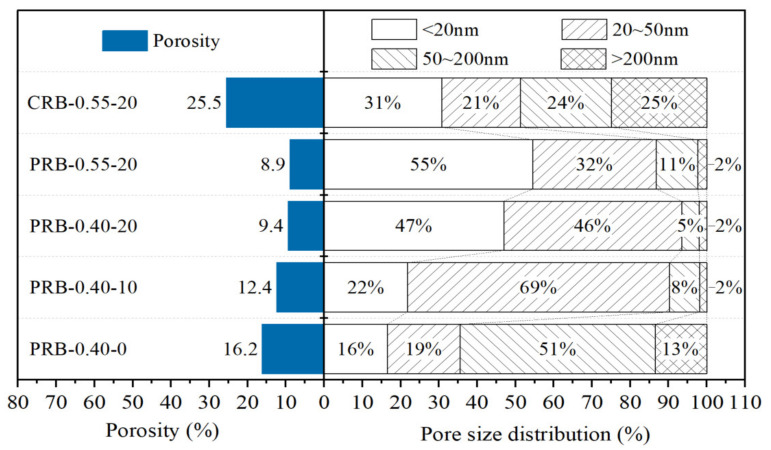
The statistical results of the porosity and pore size distribution of the pastes after 28 days of hydration.

Figure 14 presents the statistical results of the porosity and pore size distribution of the pastes. According to the degree of damage to the mechanical properties of the mortar and using the pore radius (*r*), this investigation divided the pores of the hydrated pastes into very harmful pores (*r* > 200 nm), harmful pores (50 nm < *r* < 200 nm), less harmful pores (20 nm < *r* < 50 nm), and harmless pores (*r* < 20 nm). At a fixed W/C, the addition of the BFFS could clearly reduce the porosity from 16.25% to 9.4%, while the total volume fraction of the harmless pores and the less harmful pores increased from 35% to 93%, and they then became the dominant type of the pores. When 20 vol% of the BFFS was added to the paste at a W/C of 0.55, although the total amount of the pores with a radius smaller than 50 nm was 87%, the porosity was further reduced down to 8.9%, and the volume fraction of the harmless pores increased up to 55%. The volume fractions of the harmless pores and the less harmful pores of sample CRB-0.55-20 were 31% and 21%, respectively, which indicated a potential refinement occurring in this paste due to the pozzolanic reaction of the BFFS. Figure 10 illustrates that although sample CRB-0.55-20 contained a high amount of the portlandite and bound water, a high usage of water (see Table 2) caused a high volume of the free water to remain in the hardened paste where large pores would form after its evaporation. As a result, the porosity of sample CRB-0.55-20 was calculated as 25.5%. High porosity and a high volume fraction of the harmful pores have an adverse effect on the strength of the mortar, which explains the changing patterns in Table 3.

## 4. Discussion

### 4.1. Improvement in Strength of Mortar

From an objective perspective, the efficiency of reducing the WPC consumption could not be evaluated exclusively by considering the WPC content per unit volume of the mortar. In fact, the changing pattern in the strength of the mortar should also be taken into account. When the strength of the mortar or concrete is enhanced, the construction can be designed with smaller sectional dimensions and thus less consumption of mortar or concrete, so the cement content of the mortar and energy consumption can be reduced simultaneously. Inversely, when the strength of the mortar or concrete is decreased, the construction should be designed with larger sectional dimensions and thus higher consumption of mortar or concrete, so the total cement consumption and the carbon footprint increase simultaneously. Herein, the following analysis focuses on the 3-day and 28-day flexural strength and compressive strength of the mortar mixes. 

**Figure 15:**
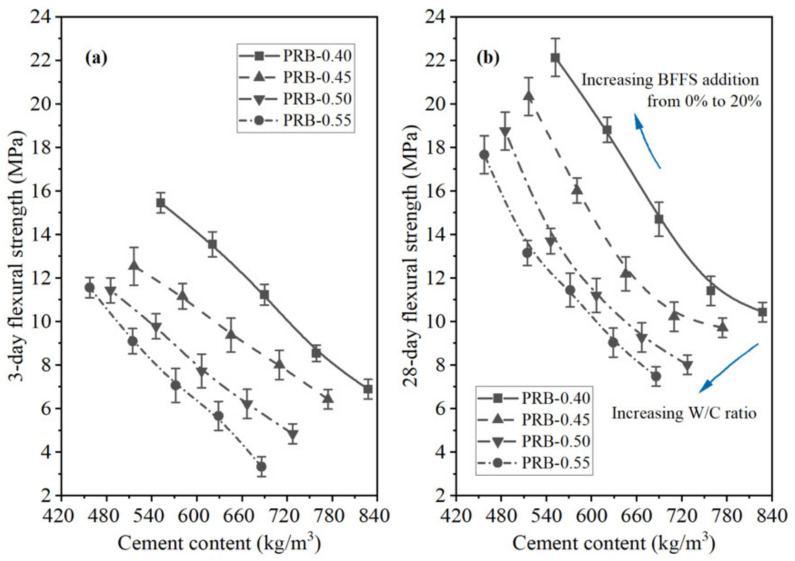
The flexural strength of the PRB mortars versus the cement content of the mortar at various water-to-cement ratios and a curing period of: (**a**) 3 days; (**b**) 28 days.

**Figure 16:**
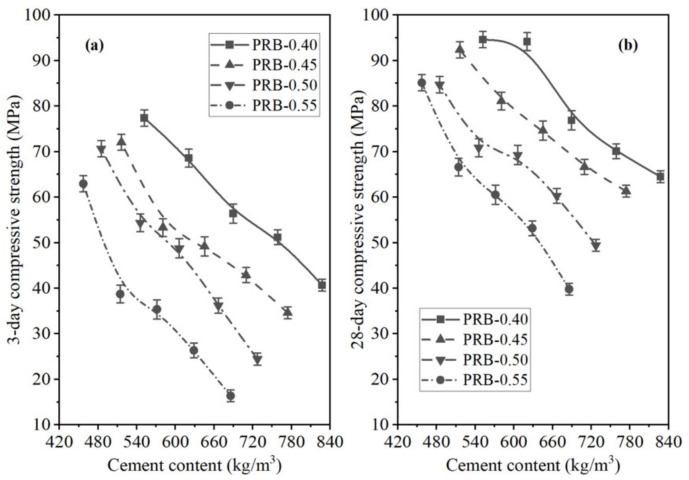
The compressive strength of the PRB mortars versus the cement content of the mortar at various water-to-cement ratios and a curing period of: (**a**) 3 days; (**b**) 28 days.

As shown in Figure 15 and Figure 16, the 3-day strength and 28-day strength of the total 20 mortar mixes designed by the paste replacement method in Table 1 are plotted against the WPC content of the mortar at different water-to-cement ratios and volumes of the BFFS. It was found that decreasing the cement content of the mortar improved the strength of the mortar both at short (3 days) and long (28 days) curing periods. These phenomena preliminarily revealed that the addition of the BFFS as a replacement for the paste not only could decrease the WPC consumption and carbon footprint but also could improve the strength of the mortar mixes. Meanwhile, raising the W/C decreased the strength of the mortar samples, but it could be compensated by increasing the volume of the BFFS in the samples with a high W/C due to the porosity refinement resulting from the pozzolanic reaction of the BFFS. For example, sample PRB-0.55-0 had the lowest 28-day compressive strength among all the mortars without the BFFS, while adding 15 vol% of the BFFS to it could raise its 28-day compressive strength to as high as 66.6 MPa (the 28-day compressive strength of sample PRB-0.55-15), which was higher than that of sample PRB-0.40-0 (64.5 MPa).

**Figure 17:**
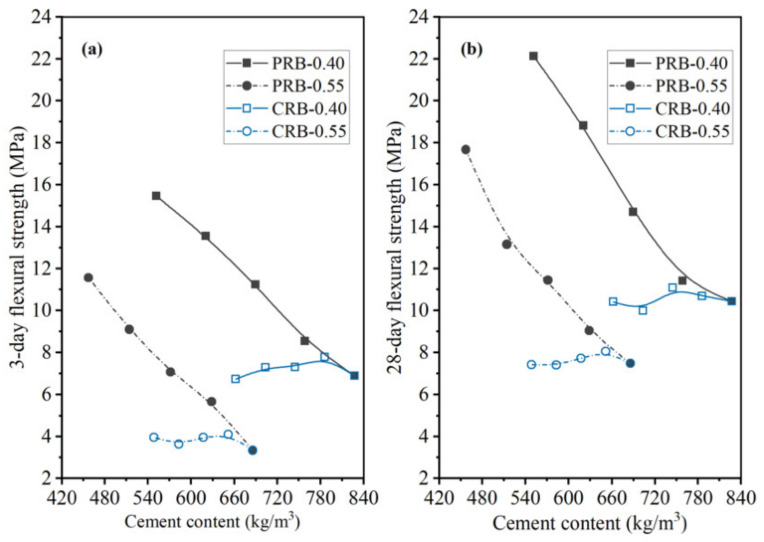
The flexural strength of the PRB and CRB mortars versus the cement content of the mortar at various water-to-cement ratios and a curing period of: (**a**) 3 days; (**b**) 28 days.

**Figure 18:**
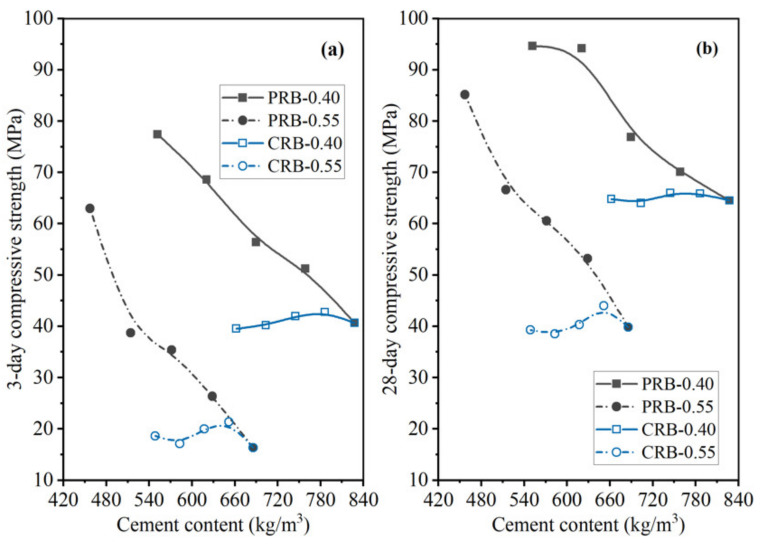
The compressive strength of the PRB and CRB mortars versus the cement content of the mortar at various water-to-cement ratios and a curing period of: (**a**) 3 days; (**b**) 28 days.

To compare the changing patterns in the strength of the PRB mortars with those in the strength of the CRB mortars, Figure 17 and Figure 18 respectively delineate the flexural strength and compressive strength of the PRB and CRB specimens at different volumes of the BFFS and water-to-cement ratios of 0.40 and 0.55. It is evident that at a given W/C, when the volume of the BFFS of the PRB mortar increased, the cement content of the mortar rapidly declined, and the flexural strength or the compressive strength of the mortar significantly improved. Nevertheless, the strength of the CRB mortar could slightly increase at 5 and 10 vol% of the BFFS but decreased at a larger volume of the BFFS, while the cement content of the mortar still remained at a high level as the volume of the BFFS rose.

**Figure 19:**
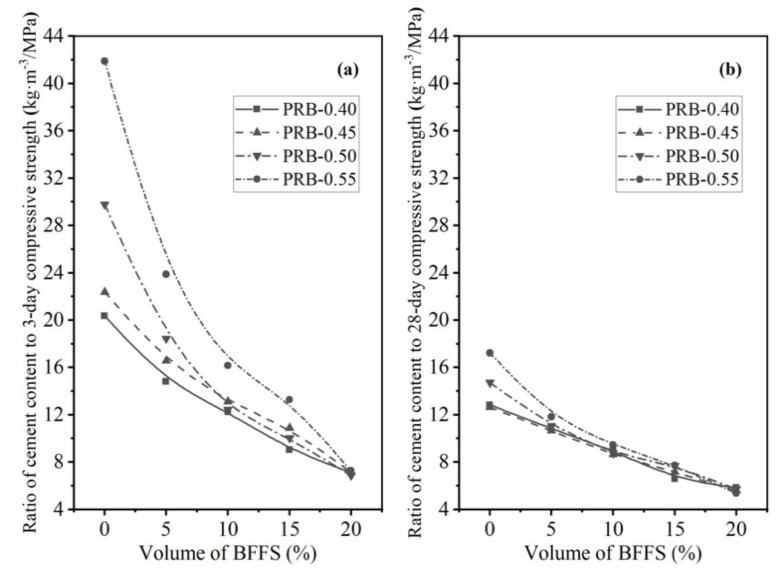
The ratio of the cement content to the compressive strength of the PRB mortars versus the volume of the BFFS at various water-to-cement ratios and a curing period of: (**a**) 3 days; (**b**) 28 days.

**Figure 20:**
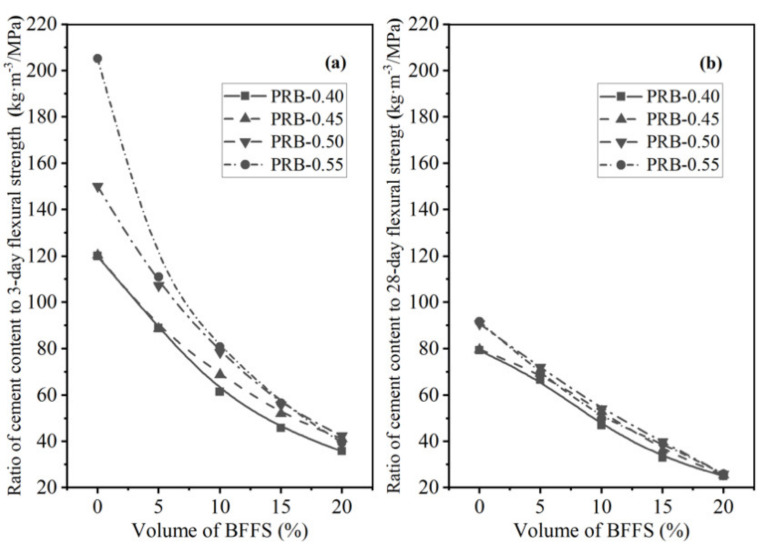
The ratio of the cement content to the flexural strength of the PRB mortars versus the volume of the BFFS at various water-to-cement ratios and a curing period of: (**a**) 3 days; (**b**) 28 days.

In addition, the present work defined two new parameters, namely the ratio of the cement content to the compressive strength (C/CS) and the ratio of the cement content to the flexural strength (C/FS), to further evaluate the synchronous changes of the flexural strength and compressive strength at different curing periods and various amounts of the WPC. According to the results of the 3-day and 28-day strength of the mortar mixes with various volumes of the BFFS, the C/CS and C/FS are plotted against the volume of the BFFS for the paste replacement method in Figure 19 and Figure 20, respectively. It can be seen in Figure 19b and Figure 20b that all the curves of the PRB samples with different water-to-cement ratios followed a monotonous decreasing trend due to the increase in the 28-day strength and the synchronous decrease in the WPC content of the mortar and finally converged to a certain point which was approximately 5.6 ± 0.2 kg·m^−3^/MPa for the 28-day compressive strength curves and 25.9 ± 0.4 kg·m^−3^/MPa for the 28-day flexural strength curves. With respect to the 3-day strength of the mortar, the monotonous trend in the convergence of the curves was more obvious in the C/CS curves in Figure 19a or C/FS curves in Figure 20a. Moreover, as the volume of the BFFS increased, the C/CS and C/FS could decrease by more than 50%. For instance, as the volume of the BFFS of sample PRB-0.40 increased from 0% to 20%, the C/CS declined from 12.8 to 5.8 kg·m^−3^/MPa (a 54.7% reduction).

**Figure 21:**
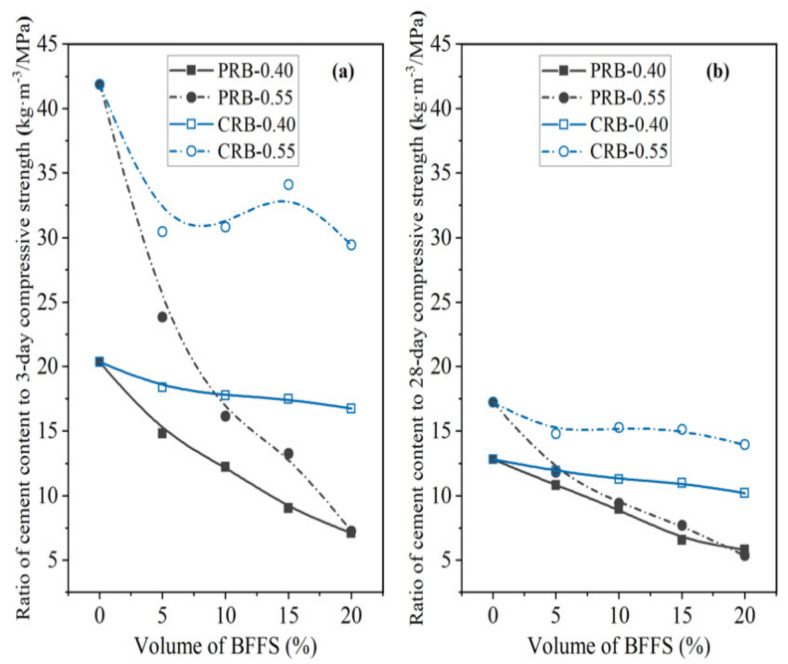
The ratio of the cement content to the compressive strength of the PRB and CRB mortars versus the volume of the BFFS at various water-to-cement ratios and a curing period of: (**a**) 3 days; (**b**) 28 days.

**Figure 22:**
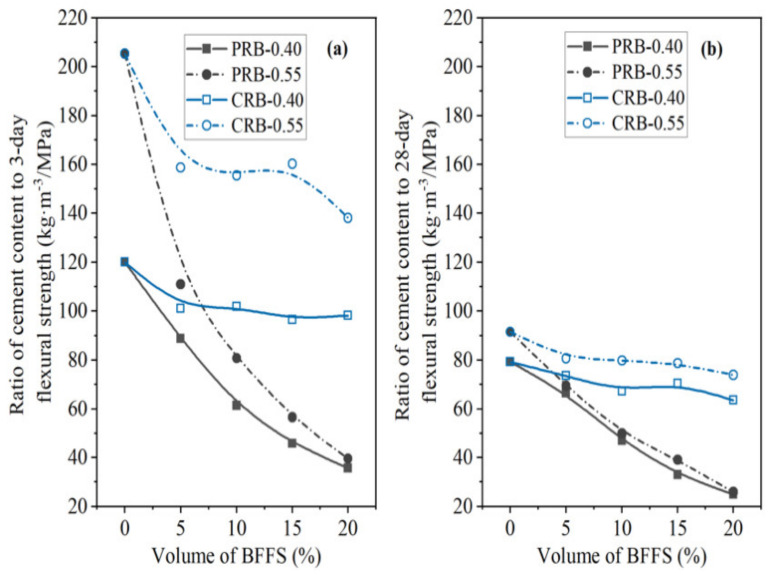
The ratio of the cement content to the flexural strength of the PRB and CRB mortars versus the volume of the BFFS at various water-to-cement ratios and a curing period of: (**a**) 3 days; (**b**) 28 days.

On the other hand, as depicted in Figure 21 and Figure 22, the incorporation of the BFFS as a replacement for the cement could not produce such a positive effect on the C/CS and C/FS since both the strength of the mortar and the WPC content of the mortar were reduced synchronously. In comparison, the paste replacement method by using the BFFS as a substitute for the paste to produce the PRB mortar with a lower ratio of the cement content to the strength was considered to be a more effective and eco-friendly strategy for utilizing the BFFS to reduce the WPC consumption and carbon footprint of mortar and to enhance its strength.

### 4.2. Roles of BFFS

On the basis of the above analysis, the roles of the BFFS as a replacement for the paste in the workability and strength of the mortar mixes can be summarized as follows:

1. As demonstrated in the above microstructure analysis, the BFFS had pozzolanic activity and could act as a supplementary cementing material in the mortar mixes, which enhanced the strength of the mortar by refining its porosity. Previous works [1,43] have also reported that the Al-to-Si ratio (Al/Si) of the samples containing the BFFS is 0.28, while that of the pure cement samples is 0.15. It appears that the amorphous alumina of the BFFS reacted at the late curing stages, and its products (mainly the C-(A)-S-H gel) mixed with the C-S-H gel from the cement hydration, which finally resulted in the improvement to the overall ratio of Al to Si. From the results of the Fourier-transform infrared (FTIR) spectroscopy [43], it could be inferred that adding the BFFS to the cement paste could shift the peak of the stretching vibration of the silicon–oxygen bond in the C-S-H gel to a higher wave number, which indicated that the degree of polymerization of the silicate chains of the C-S-H gel in the pastes containing the BFFS increased; this improvement in the degree of polymerization was also confirmed by 29Si solid-state nuclear magnetic resonance (SSNMR).
2. In addition, the fine BFFS particles could also provide nucleation sites for the precipitation of the C-S-H gel and thus could improve the hydration process of the WPC, thereby further enhancing the strength of the mortar. This nucleation phenomenon practically existed in the two different methods of recycling BFFS, but the negative impact of the additional increase in the actual W/C of the CRB samples neutralized the favorable nucleation effect of the BFFS, thereby decreasing the strength of the mortar.
3. As shown in Figure 2, the particle size distribution of the BFFS was close to that of the WPC, and the particles of both the BFFS and the WPC were finer than those of the fine aggregate; hence, the volume of the BFFS added as a replacement for the paste could increase the proportion of the powder volume (the total volume of the WPC and the BFFS). Therefore, the paste could fill the voids of the fine aggregate to improve the packing density and could coat the fine aggregate particles by forming a film, which refined the pore structure and improved the strength of the mortars.

## 5. Conclusions

To produce more eco-friendly mortar or concrete by recycling blast furnace ferronickel slag and reducing the consumption of white Portland cement, various mortar mixes with different volumes of the BFFS incorporated as a replacement either for the paste or for the cement at a series of initial water-to-cement ratios were produced for the examination of the workability of the paste and the 28-day strength of the mortar; the particle size distribution of the BFFS and the microstructure of the mortar were also analyzed. From the findings of this work, the following conclusions can be drawn:

- The BFFS chiefly consisted of SiO_2_, MgO, CaO, and Fe_2_O_3_, and its particle size distribution was continuously graded with an angular appearance.
- There was no dilution effect in the pastes produced by the paste replacement method due to the unchanged W/C. A higher dosage of the superplasticizer was also required to obtain the expected workability, which indicated the reduction in the workability of the mortars owing to the decreased water-to-powder ratio by weight.
- When the volume of the BFFS increased from 0% to 20%, the 28-day compressive strength and flexural strength of the mortar enlarged, and the 3-day strength of the mortar remarkably improved in the paste replacement method.
- The calcite of the white Portland cement reacted with the amorphous alumina of the BFFS to form the carboaluminate phase which increased the volume fraction of the hydration products and stabilized the presence of the ettringite. The XRD results proved that, at a low volume of the BFFS, monocarboaluminate was the dominant carboaluminate phase, but it was transformed into hemicarboaluminate gradually as the volume of the BFFS increased. The absence of the strätlingite was also confirmed by the XRD patterns due to the existence of the portlandite at a curing period of 28 days. The TGA results revealed that the addition of the BFFS as a replacement for the paste could consume more portlandite to produce the hydration products and form a higher amount of chemically bound water, while the pastes prepared by the cement replacement method contained high amounts of the portlandite and chemically bound water due to the dilution effect.
- The analysis of the pore structure of the mortar indicated that the paste replacement method could lead to a significant porosity refinement, and the pozzolanic activity of the BFFS could refine the pore structure of the mortar; nonetheless, there was still a great number of large pores in the pastes prepared by the cement replacement method.
- When the volume of the BFFS increased from 0% to 20%, the paste replacement method could reduce the consumption of the white Portland cement by as high as 33% and could enhance the strength of the mortars; the ratio of the cement content to the strength of the mortar also declined by at least 50%. Furthermore, the pastes were converted from a cement-based material to a BFFS-based material since the mass percentage of the BFFS in the powder material was higher than 50%. Nevertheless, in the cement replacement method, the reduction in the white Portland cement was not significant, and it was reduced by 20% at most; in addition, the strength of the mortar could only remain unchanged as the volume of the BFFS increased up to 20%.
- Compared to the cement replacement method, the paste replacement method was more effective in recycling blast furnace ferronickel slag for reducing the consumption of white Portland cement and improving the general properties of cement mortar. Therefore, it is worth further investigating this method to realize its potential adequately.

## Figures and Tables

**Table 1:** The chemical composition of the WPC and the BFFS.

Oxide	CaO	SiO_2_	Al_2_O_3_	Fe_2_O_3_	SO_3_	K_2_O	Na_2_O	MgO	Cr	Other
White Portland cement (wt %)	72.27	18.77	3.98	0.21	3.73	0.43	0.06	0.21	-	0.34
Blast furnace ferronickel slag (wt %)	31.73	26.85	20.92	1.26	4.14	0.24	0.70	9.2	1.34	3.62

**Table 2:** The mix proportions of the white mortar mix.

Sample Reference	Water (kg/m^3^)	WPC (kg/m^3^)	BFFS (kg/m^3^)	Fine Aggregate (kg/m^3^)	Dosage of SP (%)	Rate of Reduction in WPC (%)
PRB-0.40-0	331	828	0	1032	0.04	0.00
PRB-0.40-5	303	759	143	1032	0.08	8.33
PRB-0.40-10	276	690	286	1032	0.12	16.67
PRB-0.40-15	248	621	428	1032	0.23	25.00
PRB-0.40-20	221	552	571	1032	0.37	33.33
PRB-0.45-0	348	774	0	1032	0.04	0.00
PRB-0.45-5	319	710	143	1032	0.06	8.33
PRB-0.45-10	290	645	286	1032	0.10	16.67
PRB-0.45-15	261	581	428	1032	0.18	25.00
PRB-0.45-20	232	516	571	1032	0.32	33.33
PRB-0.50-0	364	727	0	1032	0.03	0.00
PRB-0.50-5	333	667	143	1032	0.04	8.33
PRB-0.50-10	303	606	286	1032	0.09	16.67
PRB-0.50-15	273	545	428	1032	0.17	25.00
PRB-0.50-20	242	485	571	1032	0.28	33.33
PRB-0.55-0	377	686	0	1032	0.00	0.00
PRB-0.55-5	346	629	143	1032	0.03	8.33
PRB-0.55-10	314	571	286	1032	0.07	16.67
PRB-0.55-15	283	514	428	1032	0.14	25.00
PRB-0.55-20	251	457	571	1032	0.23	33.33
CRB-0.40-5	331	788	38	1032	0.05	5.00
CRB-0.40-10	331	747	77	1032	0.05	10.00
CRB-0.40-15	331	706	115	1032	0.05	15.00
CRB-0.40-20	331	666	154	1032	0.06	20.00
CRB-0.55-5	377	653	32	1032	0.00	5.00
CRB-0.55-10	377	619	64	1032	0.00	10.00
CRB-0.55-15	377	585	95	1032	0.00	15.00
CRB-0.55-20	377	552	127	1032	0.00	20.00

**Table 3:** The experimental data on the slump flow and strength of the mortar specimens.

Sample ID	Slump Flow (mm)	3-Day Flexural Strength (MPa)	3-Day Compressive Strength (MPa)	28-Day Flexural Strength (MPa)	28-Day Compressive Strength (MPa)
PRB-0.40-0	210	6.9	40.7	10.4	64.5
PRB-0.40-5	210	8.6	51.2	11.4	70.1
PRB-0.40-10	195	11.2	56.4	14.7	76.9
PRB-0.40-15	330	13.6	68.6	18.8	94.2
PRB-0.40-20	280	15.5	77.4	22.1	94.6
PRB-0.45-0	200	6.4	34.6	9.7	61.3
PRB-0.45-5	190	8.0	42.9	10.2	66.7
PRB-0.45-10	195	9.4	49.2	12.2	74.6
PRB-0.45-15	345	11.2	53.3	16.0	81.1
PRB-0.45-20	315	12.6	72.1	20.3	92.4
PRB-0.50-0	265	4.9	24.4	8.0	49.4
PRB-0.50-5	210	6.2	36.2	9.3	60.3
PRB-0.50-10	220	7.7	48.8	11.2	69.3
PRB-0.50-15	335	9.8	54.4	13.7	70.9
PRB-0.50-20	302	11.4	70.7	18.8	84.7
PRB-0.55-0	240	3.3	16.4	7.5	39.8
PRB-0.55-5	210	5.7	26.3	9.0	53.2
PRB-0.55-10	260	7.1	35.4	11.5	60.6
PRB-0.55-15	325	9.1	38.7	13.2	66.6
PRB-0.55-20	335	11.6	63.0	17.7	85.2
CRB-0.40-5	230	7.8	42.8	10.7	65.9
CRB-0.40-10	220	7.3	41.9	11.1	66.0
CRB-0.40-15	235	7.3	40.2	10.0	64.0
CRB-0.40-20	218	6.7	39.5	10.3	64.8
CRB-0.55-5	225	4.1	21.4	8.1	44.0
CRB-0.55-10	215	4.0	20.0	7.7	40.3
CRB-0.55-15	200	3.6	17.1	7.4	38.5
CRB-0.55-20	225	4.0	18.6	7.4	39.3

## Data Availability

The data underlying this article will be shared on reasonable request from the corresponding author.
